# Hypercalcemia in patients with bipolar disorder treated with lithium: a cross-sectional study

**DOI:** 10.1186/2194-7511-1-18

**Published:** 2013-09-16

**Authors:** Bas A Twigt, Bernard M Houweling, Menno R Vriens, Eline J Regeer, Ralph W Kupka, Inne HM Borel Rinkes, Gerlof D Valk

**Affiliations:** Department of Surgery, University Medical Center Utrecht, Huispostnummer G04.228, P.O. Box 85500, Utrecht, 3508GA the Netherlands; Altrecht Institute for Mental Health Care, Lange Nieuwstraat 119, Utrecht, 3512 PG the Netherlands; Department of Psychiatry, VU University Medical Center, De Boelelaan 1117, Amsterdam, 1081HV the Netherlands; Department of Endocrinology, University Medical Center Utrecht, Heidelberglaan 100, Utrecht, 3583CX the Netherlands

**Keywords:** Lithium, Bipolar disorder, Hypercalcemia, Lithium-induced hyperparathyroidism

## Abstract

**Background:**

Lithium-induced hyperparathyroidism (LIH) is a relative underrecognized complication of long-term lithium treatment. Hypercalcemia may be the first, but often overlooked, sign of LIH. Symptoms of LIH can be similar to the underlying psychiatric illness, which may cause a significant doctor’s delay in diagnosing LIH. The aim of this study was to determine the prevalence of hypercalcemia in a cohort of psychiatric patients.

**Methods:**

In this cross-sectional study, we collected data from 314 patients treated with lithium in an outpatient clinic for bipolar disorder. Patients with bipolar disorder from the same clinics, who had never been treated with lithium and of whom serum calcium levels were available, were included as controls (*n* = 15). Patient characteristics and laboratory results were collected during the period of June 2010 till June 2011.

**Results:**

The mean serum calcium level was 2.49 (SD 0.11) mmol/l. The point prevalence of hypercalcemia (>2.60 mmol/l) was 15.6%. In a comparable group of psychiatric patients not using lithium, the mean serum calcium level was 2.37 mmol/l, and none of these patients had hypercalcemia (*p* = 0.001). The duration of lithium treatment was the only significant predictor for the development of hypercalcemia (*p* = 0.002).

**Discussion:**

The prevalence of hypercalcemia in lithium-treated patients was significantly higher than that in non-lithium treated controls and correlated to the cumulative time lithium was used in this cross-sectional study. We recommend that serum calcium levels should be routinely tested in patients using lithium for timely detection of LIH or hypercalcemia due to other causes.

## Background

Lithium is used effectively in the treatment of bipolar disorder over the last 60 years, and despite the availability of many pharmacotherapeutic options, it remains the cornerstone of long-term prophylaxis (Cade 1949). Hypercalcemia associated with lithium-induced hyperparathyroidism (LIH) is a common, but easily overlooked, complication of lithium treatment. Monitoring of serum calcium levels is therefore recommended in the International Society for Bipolar disorders (ISBD) consensus guidelines for the safety monitoring of bipolar disorder treatments since 2009 (Ng et al. 2009). The first case of LIH was published in 1973 (Garfinkel et al. 1973). The reported prevalence of hypercalcemia in patients on lithium therapy varies among studies from 15% to 60% (Bendz et al. 1996; Ananth and Dubin 1983; Davis et al. 1981; Järhult et al. 2010; Saunders et al. 2009; Christensson 1976; Szalat et al. 2009; Khandwala and Van Uum 2006).

Since symptoms of LIH such as fatigue, weakness, and depression can be similar to the symptoms of the mood disorder for which lithium therapy was initiated, this may cause a significant doctor’s delay in the diagnosis of LIH when misinterpreted (Khandwala and Van Uum 2006; Houweling et al. 2012; Rizwan and Perrier 2009). The exact pathophysiology by which LIH develops is still unknown. Lithium may induce LIH directly or only unmask or accelerate a previously unnoticed hyperparathyroidism (Birnbaum et al. 1988; Tupin et al. 1968; Gerner et al. 1977; Mellerup et al. 1976; Saxe and Gibson 1991; Saxe et al. 1995). There are no clear predictors to determine which patients are at risk for LIH. Furthermore, the way in which these patients should be treated is still a matter of debate. Discontinuation of lithium treatment, if possible, has been proven unsuccessful in most patients that have been using lithium for longer periods (Garfinkel et al. 1973; Bendz et al. 1996; Bendz et al. Järhult et al. 2010; Saunders et al. 2009; Houweling et al. 2012). Different surgical approaches of parathyroidectomy have been described, but calcimimetics or a ‘wait and see policy’ might be an alternative to surgery (Bendz et al. 1996; Ananth and Dubin 1983; Davis et al. 1981; Järhult et al. 2010; Saunders et al. 2009; Christensson 1976; Szalat et al. 2009; Khandwala and Van Uum 2006; Houweling et al. 2012; Hundley et al. 2005; Sloand and Shelly 2006; Gregoor and de Jong 2007).

Given the unfamiliarity with this condition, periodical monitoring of serum calcium has only recently been implemented in psychiatric guidelines and is still less well-known in comparison to other well-known complications regarding thyroid and kidney function (Ng et al. 2009; Van de Beek et al. 2010). We therefore assume that LIH is both underdiagnosed and undertreated.

The aim of this study was to determine the prevalence of hypercalcemia in a large sample of outpatients treated with lithium for bipolar disorder. To gain insight into which of these patients are more prone to develop LIH, we assessed which determinants were related to the development of hypercalcemia.

## Methods

### Patients and controls

The medical records of patients with bipolar disorder who were treated with lithium between June 2010 and June of 2011 at an outpatient clinic for bipolar disorder were reviewed. Inclusion criterion was the continuous use of lithium and at least one measurement of the serum calcium level. Outpatients with bipolar disorder from the same clinics, who had never been treated with lithium and of whom serum calcium levels were available, were included as controls.

### Outcomes

The following data were collected from the medical records: age, gender, past medical history, medication current lithium dose, any possible interruptions during the period that lithium was taken, and total cumulative duration of lithium treatment. The following laboratory variables were registered and analyzed by routine methods (reference values between brackets): serum levels of lithium (0.6 to 1.2 mmol/l), calcium (2.10 to 2.55 mmol/l), parathormone (PTH) (1.0 to 7.0 pmol/l), albumin (38 to 42 mmol/l), creatinine (74 to 110 μmol/l), thyroid-stimulating hormone (TSH) (0.35 to 5.00 mU/l), FT4 (9 to 27 pmol/l), sodium (135 to 145 mmol/l), and potassium (3.5 to 5.0 mmol/l). Also, any medication used by the patient was registered since thiazide/chloortalidon diuretics, calcimimetics, vitamin D supplements, and calcium supplements can induce increased serum calcium levels.

### Data analysis

The Kolmogorov-Smirnov test was employed to test for normal variance for each variable. Statistical differences were calculated by means of the Student’s independent *t* test and Pearson’s chi-square test. The missing data resulted in a lower *n* than the actual number of patients. Statistical significance was set as a two-tailed *p* < 0.05. PASW Statistics IBM version 18 (IBM, New York, USA) was used.

### Definitions

The point prevalence of hypercalcemia was defined as the prevalence of elevated serum calcium levels within our cross-sectional 1-year time frame. If more than one serum calcium level was available, the average serum calcium level was determined.

The upper limit for serum calcium was set at 2.60 mmol/l. The total duration of lithium use was defined as the cumulative period that lithium was taken by a patient expressed in months. If the use of lithium had been interrupted, the total duration of lithium reflects the sum of all individual periods.

### Ethical committee

Approval was obtained from the medical ethics committee of the University Medical Centre Utrecht (registration protocol 10/340C).

## Results

### Descriptive analysis

A total of 314 patients were identified who were currently using lithium and in which the serum calcium level was determined at least once. The control group consisted of 15 patients with bipolar disorder who had never used lithium and from whom a serum calcium measurement was available.

### Patients on lithium

In the 314 lithium-treated patients, the mean age was 47 years and 61.5% were women (*n* = 195). The mean dosage of lithium was 960 (SD 280) mg a day. The mean serum level of lithium was 0.74 (SD 0.19) mmol/l. The mean period that lithium was taken was 117.6 (SD 91.2) months, and the point prevalence of hypercalcemia was 15.6% (*n* = 49). None of these patients had a medical history of hyperparathyroidism, hypercalcemia, or kidney failure, nor did they use any medication with hypercalcemia as a possible side effect.

**Figure 1:**
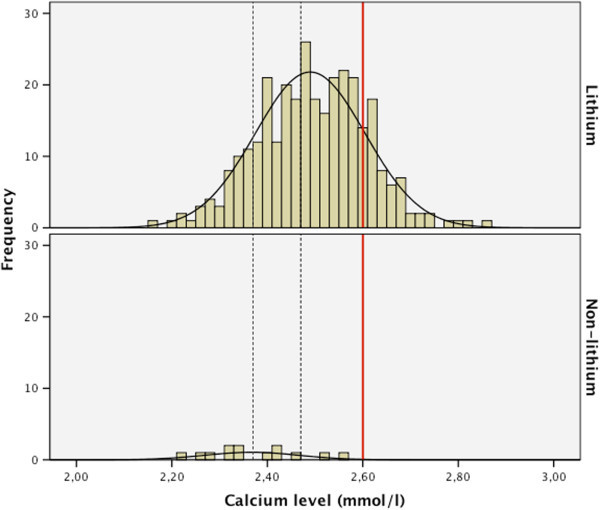
**Distribution curves of serum calcium levels in lithium users (** ***n*** **= 314) and non**-**lithium users (** ***n*** **= 15).** The red line illustrates the upper limit of the serum calcium level of 2.60 (mmol/L). The two dotted lines are placed upon the mean serum calcium levels of both groups; the left dotted line corresponds with the non-lithium users, the right dotted line with the lithium users.

In the control group, the mean serum calcium level was 2.37 (SD 0.10) mmol/l, which was significantly (<0.001) lower compared to that of the lithium users (Table 1). None of these patients had a serum calcium level above 2.60 (mmol/l), as illustrated in Figure 1.

**Table 1:** **Differences between lithium users and non**-**lithium users**

	Lithium users (***n*** = 314)	Non-lithium users (***n*** = 15)	***p*** value
Gender (% female)	61.5	66.7	0.934
Age (years)	47 (13)	40 (9)	0.036
Creatinine (μmol/l)	77.59 (18.9)	68.45 (14.8)	0.115
TSH (mU/l)	2.7 (3.6)	1.9 (0.7)	0.454
Sodium (mmol/l)	140.5 (2.26)	140.9 (1.30)	0.353
Potassium (mmol/l)	4.5 (0.35)	4.3 (0.26)	0.025
Calcium serum level (mmol/l)	2.49 (0.11)	2.37 (0.10)	<0.001

Mean values and standard deviation (SD) between brackets. Statistical differences were calculated by means of the Pearson’s chi-square test (gender) and Student’s independent *t* test (all other variables).

### Subgroup analysis

Subgroup analysis showed that the cumulative duration of lithium use (i.e., the total time that a patient had used lithium regardless of any possible interruptions) was the only predictor for the development of hypercalcemia. The mean cumulative duration of lithium use was 165 months in hypercalcemic patients versus 109 months in patients with normal calcium levels (*p* = 0.002).

In addition, we calculated a hypothetical lifetime lithium dose by multiplying the cumulative duration of lithium use with the most recent dose. This was significantly different between both groups (*p* = 0.002). All other factors such as gender (*p* = 0.457), current lithium dose (*p* = 0.759), serum lithium level (*p* = 0.379), age (*p* = 0.174), serum creatinine (*p* = 0.091), and thyroid function (*p* = 0.176) did not differ significantly (Table 2).

**Table 2:** **Subgroup analysis of lithium users with normal serum calcium levels compared to lithium users with hypercalcemia**

	Lithium users with serum calcium level ≤2.60 (mmol/l) (***n*** = 265)	Lithium users with serum calcium level >2.60 (mmol/l) (***n*** = 49)	***p*** value
Gender (% female)	60.4	67.3	0.457
Age (years)	47 (13)	50 (13)	0.174
Lithium recent dosage (mg dd)	960 (270)	970 (330)	0.759
Creatinine (μmol/l)	76.82 (17.00)	81.91 (26.89)	0.091
TSH (mU/l)	2.8 (3.8)	2.0 (1.8)	0.176
Sodium (mmol/l)	140.5 (2.20)	140.7 (2.61)	0.610
Potassium (mmol/l)	4.5 (0.34)	4.5 (0.37)	0.349
Lithium serum level (mmol/l)	0.74 (0.19)	0.77 (0.18)	0.379
Lithium period (months)	109 (84)	165 (111)	0.002
Lifetime dosage quotient	1.03 × 10^5^ (8.3 × 10^4^)	1.63 × 10^5^ (1.3 × 10^5^)	0.002

Mean values and standard deviation (SD) between brackets. Statistical differences were calculated by means of the Pearson’s chi-square test (gender) and Student’s independent *t* test (all other variables).

### Hypercalcemic patients

Of the 49 patients with calcium levels above 2.60 mmol/l, 3 patients were referred to an endocrine physician or surgeon to reveal the cause of hypercalcemia. Two of these patients with serum calcium levels of 2.71 and 2.65 mmol/l and elevated PTH levels of 17.2 and 53.6 pmol/l, respectively (reference value 1.3 to 9.3 pmol/l), underwent minimally invasive parathyroidectomy after preoperative work-up confirmed the diagnosis of LIH. The third patient was treated with cinacalcet since the preoperative work-up showed inconsistent results regarding the presence and location of any adenoma. The other 46 patients remained under strict control, i.e., repeated measurements of the serum calcium levels and PTH levels at least twice a year as directed by the ISBD consensus guidelines referred to earlier, but were at the time of this cross-sectional study not referred for further analysis (Ng et al. 2009). Follow-up is currently conducted to obtain better insight into the clinical course of these patients.

## Discussion

### Main findings

In this cross-sectional study, we found a significant higher prevalence of hypercalcemia in patients using lithium compared to that of the control group of patients without a current and previous history of lithium treatment (*p* = 0.001). In addition, the development of hypercalcemia was correlated to the cumulative time that lithium was taken regardless whether this was continuous or with one or more interruptions of various durations.

### Strengths and weaknesses

This study is the first to determine the prevalence of hypercalcemia in a major cohort of psychiatric patients. Our results regarding the prevalence corroborates with previous studies (Bendz et al. 1996; Ananth and Dubin 1983; Davis et al. 1981; Järhult et al. 2010; Saunders et al. 2009; Christensson 1976; Hundley et al. 2005; Awad et al. 2003; Carchman et al. 2008). However, compared to previous studies, in our study, we included a large number of patients, and selection bias is less likely because we used a predefined search for patients that were currently treated in a large, non-academic psychiatric outpatient clinic. Patients attending this clinic reflect a regular sample of bipolar outpatients of varying severity and duration of illness. In most other studies, patients were selected from an endocrine surgery database instead of a psychiatric cohort. As a consequence, these cohorts included a highly selected subgroup of lithium-treated patients since the outcome, i.e., hyperparathyroidism, was already present.

In our study, patients were identified based upon their bipolar disorder, and calcium levels were measured as part of a routine monitoring schedule. In addition, using an upper limit for serum calcium levels of 2.60 mmol/L compared to that of the various lower upper limits uphold among different hospitals, psychiatric facilities, and laboratories, it is unlikely that our results overestimate the prevalence of hypercalcemia. All other factors such as medical history, use of other medications besides lithium, and possibly previous surgery were taken into account. None of these showed a significant difference that could distort our results.

Although our control group of lithium-naïve patients was very small compared to the group of lithium users reflecting the widespread use of lithium for bipolar disorder in the Netherlands, the range from low to high calcium levels seems to be higher in lithium users than that in the control patients. This may be an indication that the set point of serum calcium is higher in lithium users due to the binding of lithium to the calcium-sensing receptor of the parathyroid cell. This binding results in a lower threshold for the secretion of PTH, implicating that the serum calcium is also higher in all lithium users, and leading to hypercalcemia in approximately 15%. Our control group was small (*n* = 15), and therefore, such conclusions remain preliminary. Nonetheless, this cross-sectional study is the first including a control group of psychiatric patients, in this perspective. Because lithium is the mainstay for the long-term treatment of bipolar disorder, it is difficult to identify a larger cohort of control patients.

Whether lithium shifts the calcium set point to the right or that it uncovers a preexisting hyperparathyroidism remains the question. Because of the absence of PTH measurements, calcium in 24-h urine specimens, and proper imaging of patients with suspected LIH, we were unable to investigate this in our sample, which poses a limitation of our study.

### Treatment

In the absence of an evidence-based consensus on the optimal surgical approach in lithium-induced hyperparathyroidism, several studies have been published regarding the different treatment options (Bendz et al. 1996; Järhult et al. 2010; Saunders et al. 2009; Abdullah et al. 1999; Awad et al. 2003; Carchman et al. 2008). Some advocate removal of all four glands where others apply a more focused approach. In order to determine the optimal treatment for LIH, one has to understand its pathophysiology. However, the underlying mechanism by which lithium induces hyperparathyroidism has not been elucidated yet. Since lithium was thought to induce hyperplastic changes to all four glands (based on the idea that all glands are exposed equally) rather than the formation of one or multiple adenomas, (Abdullah et al. 1999), routine complete neck exploration was the surgical procedure of choice for a long time. The overall prevalence of multiglandular disease, however, has a broad variance ranging from only 13% to 52% (Järhult et al. 2010; Abdullah et al. 1999; Awad et al. 2003; Carchman et al. 2008). And therefore, the notion that the remaining parathyroid glands will render pathological with time is perhaps premature and lacks sufficient evidence (Järhult et al. 2010; Saunders et al. 2009). This would support a more selective approach and more extensive preoperative imaging as is common nowadays in primary hyperparathyroidism. At this moment, it is too early to advocate a certain surgical approach since both theories find equal supports in the literature. Furthermore, when there is reluctance to surgery, a relative new class of drugs called calcimimetics has been shown to lower the serum calcium level. Even though the results are promising, one must realize that this is not a definitive therapy (Houweling et al. 2012; Rothe et al. 2011).

## Conclusions

The present study clearly established that the prevalence of hypercalcemia is significantly higher in patients using lithium in a major cohort of patients. Furthermore, our subgroup analysis showed that the total duration of lithium treatment was significantly longer in the hypercalcemia group, regardless of any possible interruptions or discontinuation of lithium treatment.

Our results underscore that it is mandatory to perform regular monitoring of serum calcium as well as PTH levels. LIH cannot be diagnosed based upon signs and symptoms alone, since these are easily misinterpreted in the context of mood disorders. With the growing awareness of LIH, future research should focus on the best treatment for this delicate group of patients.
